# The impact of the transversus abdominis plane block (TAP) on stress response measured through the complete blood– derived inflammatory markers

**DOI:** 10.1007/s11259-023-10234-7

**Published:** 2023-10-16

**Authors:** Lorena Espadas-González, Jesús M. Usón-Casaús, Nieves Pastor-Sirvent, Massimo Santella, Javier Ezquerra-Calvo, Eva M Pérez-Merino

**Affiliations:** 1 Unidad de Cirugía, Departamento de Medicina Animal, Facultad de Veterinaria UEx, Universidad de Extremadura, Avenida de la universidad s/n, Cáceres, 10003 Spain; 2 Veterinary Teaching Hospital, Facultad de Veterinaria, Universidad de Extremadura, Avenida de la universidad s/n, Cáceres, 10003 Spain

**Keywords:** Transversus Abdominis plane block, Neutrophil– to– lymphocyte ratio, Platelet– to– lymphocyte ratio, Systemic immune– inflammation index, Inflammation, Laparoscopy

## Abstract

This study aims to evaluate the effect of the transversus abdominis plane (TAP) block on the blood cells and the inflammatory markers neutrophil– to– lymphocyte ratio (NLR), platelet– to– lymphocyte ratio (PLR), and systemic immune– inflammation index (SII) after the laparoscopic ovariectomy (LapOV) in dogs. 72 healthy bitches undergoing LapOV were randomly allocated to the no– TAP group of dogs under inhaled anesthesia (IA), the TAP– S group (IA and TAP with saline), and the TAP– B group (IA and TAP with bupivacaine). The NLR, PLR, and SII were calculated 1 h before ovariectomy (T0) and at 2−3 h (T1), 6−8 h (T2), and 20−24 h (T3) post– surgery. The number of dogs requiring postoperative analgesic rescue with buprenorphine and the doses administered in each group were recorded. Significant changes were observed in all groups’ postoperative NLR, PLR, and SII over time. Between groups, no differences were observed in any of the ratios at any control point (NLR at T0−T3: *p* = 0.17, 0.36, 0.80, and 0.95; PLR at T0−T3: *p* = 0.70, 0.62, 0.21, 0.87; SII at T0−T3: *p* = 0.29, 0.65, 0.09, and 0.34). A significantly lower number of dogs required analgesic rescue in the TAP-B group (*p* = 0.0001) and a lower number of doses were administered (*p* = 0.001). There is no difference in the inflammatory response measured through the complete blood– derived inflammatory markers after the LapOV in dogs when the postoperative pain is managed entirely with opioids or with the TAP block with bupivacaine. The hydrodissection associated with the TAP block technique does not increase the inflammatory response.

## Introduction

The stress response to surgery is affected by several factors related to the procedure, patient, and postoperative care. It is well– known that anesthesia itself suppresses the immune system, influencing the functions of immunocompetent cells and inflammatory mediator gene expression and secretion (Kurosawa and Kato 2008). Therefore, different studies have focused on the endocrine and metabolic stress responses to surgery by different anesthesia techniques (Saunders et al. 2009; Tomihari et al. 2015; Fujiyama et al. 2021; Imani Rastabi et al. 2021).

Complete blood– based inflammatory markers, such as the neutrophil– to– lymphocyte ratio (NLR), the platelet– to– lymphocyte ratio (PLR), and the systemic immune– inflammation index (SII), are simple and inexpensive markers of the inflammatory response. In humans, some of these markers have been proven to be affected not only by surgical trauma but also by the anesthetic method (Kim et al. 2011; Alkan et al. 2018; Ni Eochagain et al. 2018; Erbaş et al. 2019; Surhonne et al. 2019; Tanriverdi et al. 2021). Specifically, some of those studies have demonstrated the benefits of spinal anesthesia over general anesthesia in terms of lower NLR after surgery (Alkan et al. 2018; Erbaş et al. 2019; Surhonne et al. 2019). However, no study in veterinary medicine evaluates the effects of different anesthetic techniques on these markers.

The transversus abdominis plane (TAP) block is an anesthetic technique of great interest for locoregional anesthesia in veterinary practice. This block is becoming increasingly popular in procedures with modest/high pain, such as canine ovariectomy, ovariohysterectomy, and mastectomy (Cicirelli et al. 2022). Recently, the use of the TAP block in bitches undergoing laparoscopic ovariectomy (LapOV) has been proven to diminish the signs of intra– operative nociception and postoperative pain, reducing the isoflurane concentration during the anesthesia and the postoperative opioid requirements (Espadas-González et al. 2022; Paolini et al. 2022).

However, the puncture and the hydrodissection of the muscular plane needed to place the local anesthetic during the TAP procedure, and the local anesthetic– induced tissue damage might cause additional soft tissue trauma, increasing the inflammatory response. Even so, studies in humans have attributed the immunomodulatory effects of the TAP block to the ability to reduce pro– inflammatory cytokines (Canakci et al. 2021) and the stress response (Qin et al. 2019). Nevertheless, the effect of the TAP block on the complete blood– derived inflammatory markers has never been studied in veterinary patients.

Therefore, this study aimed to evaluate whether the TAP block might influence the inflammatory response measured through the NLR, PLR, and SII in dogs undergoing one of the most common routine elective surgeries, the LapOV. Secondarily, we also investigated the changes in the blood cells that led to the modifications in the markers.

## Methods

This prospective, randomized clinical study was conducted at the Veterinary Teaching Hospital of the University of Extremadura under the approval of the University of Extremadura Animal Care and Use Committee (register number 168/2021, approved December 15, 2021). Written informed consent was obtained from the manager of the animal shelter from which the bitches came.

The inclusion criteria for this study were healthy female dogs (American Society of Anesthesiologists [ASA] physical status I) based on physical examination and clinical laboratory data: complete blood count and serum biochemical analysis (determination of total protein, albumin, alanine aminotransferase, aspartate aminotransferase, alkaline phosphatase, gamma-glutamyl transpeptidase, total bilirubin, cholesterol, urea nitrogen, creatinine, glucose, phosphorus, calcium, chloride, potassium, and sodium serum concentrations) admitted for a neutering procedure. Exclusion criteria included being in the shelter for less than a month, aged < 6 months, pregnancy, any signs of systemic disease, cardiac or respiratory alteration, and baseline hematological values outside the normal range. For Greyhounds, breed– specific reference intervals were used to identify abnormalities in the blood tests (Campora et al. 2011).

Patients were randomly distributed into three groups depending on the anesthetic protocol: a group of dogs was assigned to receive general inhalation anesthesia (no– TAP group), another to general inhalation anesthesia combined with TAP block with bupivacaine (TAP– B group), and the third group to inhaled anesthesia and TAP block with saline solution (TAP– S group). Each dog was assigned a number by an employee of the animal shelter blinded to the study groups, and computer–generated lists of randomized numbers (www.randomizer.org) were used to allocate dogs to each group.

The sample size was estimated based on the previous calculations performed by Kim et al. (2011). According to previous results (Kim et al. 2011; Alkan et al. 2018; Ni Eochagain et al. 2018), a significant effect size was determined as a difference of NLR of 20% between the groups after surgery. With an alpha value of 0.05 and a power of 0.8, a sample size of 20 dogs per group was required. To compensate for possible dropout, 26 patients were enrolled in each no– TAP and TAP– B groups, while the number of dogs in the TAP– S group was kept to the minimum (n = 20) due to ethical considerations.

The anesthesiologist that explored the patients preoperatively, prepared the solutions, induced the dogs, and performed the TAP was different from the anesthetist that monitored the anesthesia and the postoperative period and obtained postoperative blood samples, who was blinded to the treatment group. The laboratory staff was also blinded to the dog group.

Preoperative food fasting of 8−12 h was observed.

### Surgical procedure

All dogs underwent a midline– three– port LapOV. Pneumoperitoneum was created using a Veress technique, and abdominal pressure was maintained at 8−10 mmHg. Two 5– mm portals were placed on the midline, in the middle and cranial position, and a 5– or 3– mm trocar was inserted in the caudal position depending on the dog’s size. The animal was positioned with lateral tilt, and the ovary was suspended with laparoscopic Allis grasping forceps (Clickline®, Karl Storz Endoscopy, Tuttlingen, Germany). The LigaSure™ (Medtronic, Minneapolis, MN, USA) vessel– sealing device was used to coagulate and cut the suspensory ligament, ovarian pedicle, and fallopian tube. The procedure was repeated at the contralateral side, and both ovaries were removed through the middle or caudal cannula or the abdominal incision. Finally, the pneumoperitoneum was released, and the portals were closed with 2/0 polyglyconate sutures (Monosyn®, B Braun, Tuttlingen, Germany) for inner layers and 3/0 for intradermal sutures.

### General anesthesia

Premedication consisted of a combination of methadone (Semfortan® 10 mg mL^−1^, Eurovet Animal Health BV, Bladel, Netherlands) at 0.3 mg kg^−1^ and dexmedetomidine (Dexmopet® 0.5 mg mL^−1^, Vetpharma Animal Health SL, Barcelona, Spain) at 3 mcg kg^−1^ intramuscular. Fifteen minutes after premedication and if a mild– to– moderate degree of sedation was obtained (Gurney et al. 2009), the cephalic vein was catheterized for the administration of fluids (Ringer’s lactate solution 5 mL kg ^−1^ h^−1^) and drugs.

Administration of intravenous propofol (Propofol– Lipuro® 10 mg mL^−1^, B. Braun VetCare SA, Barcelona, Spain) prior to endotracheal intubation was performed by manual injection, starting from a standard estimated dose of 4 mg kg^− 1^, which was administered in increments of 1 mg/kg over 30 s, followed by a pause of 30 s after which intubation conditions were assessed (loss of medial palpebral reflex and of jaw tone before performing endotracheal intubation.). If conditions for tracheal intubation were not adequate, a further 1 mg/kg increment of propofol was administered over 30 s, followed by a 30-second pause.

Following endotracheal intubation, isoflurane (IsoFlo®, Zoetis Spain SL, Alcobendas, Madrid) in 100% oxygen was administered through an appropriate breathing system. All dogs were mechanically ventilated using a pressure control mode to maintain end– tidal CO_2_ between 35 and 45 mmHg, with a maximum peak inspiratory pressure of 15 cm H_2_O and inspiratory:expiratory ratio of 1:3. Dogs were monitored using a multiparametric monitor (CARESCAPE™ Monitor B650, GE Healthcare, Helsinki, Finland). Parameters monitored included heart rate, temperature, end– tidal CO_2_, arterial hemoglobin saturation, end– tidal isoflurane concentration, tidal volume, compliance, cranial reflexes, and non– invasive blood pressure (oscillometric method). In addition, before surgery, the dorsal metatarsal artery was percutaneously catheterized for invasive blood pressure monitoring. This catheter was removed before anesthesia recovery. All physiological data were recorded every 5 min. If during the surgical procedure the anesthetist observed signs of a sudden nociceptive response to surgery, a 0.5 mg/kg IV rescue bolus of propofol was administered (Gomes et al. 2020; Lardone et al. 2022).

At the end of the surgery, isoflurane and fluid therapy were discontinued in both groups, and dogs were extubated when the swallowing reflex returned.

### TAP block technique

To perform the bilateral ultrasound guided– TAP block, the procedure described by Espadas-González et al. (2022) was followed. The same experienced anesthetist performed all the blocks. Dogs were placed in dorsal recumbency with the abdominal hair previously clipped and the skin aseptically prepared.

A linear 11.7– MHz ultrasound probe attached to an ultrasound machine (GE Logiq S7 Expert, GE Healthcare, Little Chalfont, UK) was firstly positioned parallel to the caudal border of the last rib (subcostal approach) and, secondly, cranial to the crest of the ilium (umbilical approach) with a transverse orientation. After the correct visualization of the three abdominal muscle layers (external abdominal oblique, internal abdominal oblique, and transversus abdominis), a 22-gauge (88 mm) or 22‐gauge (35 mm) Quincke spinal needle (Spinocan®, B. Braun, Recklinghausen, Germany) was inserted into the plane under ultrasound guidance and advanced in a cranial– to– caudal direction for the subcostal approach and in a ventral– to– dorsal direction for the umbilical approach until the tip was visualized between the rectus abdominis and the transversus abdominis muscles for the subcostal approach and between the obliquus internus and transversus abdominis muscles for the umbilical approach. The needle was connected to an extension set and a syringe (BD Discardit™ II, Becton Dickinson SA, Madrid, Spain). Hydrodissection of the correct muscular plane was confirmed by injecting a small amount of the solution. If the ultrasound imaging suggested that the location was incorrect and hydrodissection was not observed, the needle was repositioned, and the test injection was repeated. When satisfactory needle positioning was achieved, the remaining volume of the solution was injected, leading to TAP expansion, which appeared as a hypoechoic space.

A total volume of 0.3 mL kg^−1^ of 0.9% sodium chloride (B. Braun Vet Care, Milano, Italy), including a dose of 0.5 mg kg^−1^ of bupivacaine 0.5% (B. Braun, Rubí, Barcelona) was administered at each injection site in the TAP– B group, and the same volume of only saline solution was injected in the TAP– S group. In the TAP– B group, 10 min of standby time was set between the end of the TAP procedure and the beginning of the surgery.

### Postoperative follow– up and parameters assessed

All dogs were hospitalized for 24 h after surgery. During the postoperative period, buprenorphine (Buprecare® 0.3 mg mL^−1^, Ecuphar NV, Oostkamp, Belgium) at a dose of 15 mcg kg^−1^ was administered intravenously as rescue analgesia if the evaluator recorded scores were above five on the Glasgow Composite Pain Measurement Scale (CMPS) (Holton et al. 2001; Reid et al. 2007), above five on the Melbourne Pain Scale (MPS) (Firth and Haldane 1999), or above three on the Colorado Pain Scale (CPS) (Pelligand and Sanchis 2016). The number of dogs requiring rescue analgesia was recorded for each group.

The age, weight, and breed of all study patients were documented. The anesthetic time from the intubation to the end of the isoflurane administration, and the surgical time, defined as the time between the Veress’ needle insertion and the end of the skin closure, were recorded.

Blood samples (4.5 mL) were collected by jugular venipuncture 1 h before ovariectomy (T0) and at 2−3 h (T1), 6−8 h (T2), and 20−24 h (T3) post– surgery. A hematological study with white blood cell (WBC) differential was performed using an IDEXX ProCyte Dx Analyzer, (IDEXX B. V., Hoofddorp, The Nederlands), and the absolute platelet, neutrophilic, and lymphocytic counts were recorded.

NLR and PLR were calculated by dividing the absolute neutrophil (×10^9^) and platelet number (×10^9^), respectively, by the absolute number of lymphocytes (×10^9^). SII was calculated by the formula: platelet count × [neutrophil count / lymphocyte count].

NLR, PLR, and SII were determined for each dog at T0, T1, T2, and T3.

### Statistical analysis

Data were analyzed using the Statistical Package for the Social Sciences (IBM SPSS for Windows, version 27.0; IBM Corp., Armonk, NY, USA). Data distribution was assessed using the Shapiro–Wilk test. Normally distributed data are presented as mean (standard deviation), whereas the non– normally distributed values are expressed as median (range). Kruskal−Wallis and Dunn post hoc tests were used to compare the age, weight, surgical and anesthetic time, analytes absolute counts, NLR, PLR, and SII between the groups. Categorical variables such as the incidence of the need for rescue analgesia, were compared with the χ2 test or Fisher exact test as appropriate. Dogs that required analgesic rescue were not excluded from statistical analysis.

Friedman’s test was used to compare the values of the ratios among the different time points within the same group, followed by the Wilcoxon paired test if differences were observed. For all variables, the differences were considered statistically significant when *p* < 0.05.

## Results

### Perioperative parameters

A total of 80 female dogs scheduled for LapOV were enrolled in the study. Of these, three were excluded due to the presence of large ovaries that required considerable enlargement of the trocar incision for their removal or to findings that led to deciding to perform ovariohysterectomy instead of ovariectomy and two because technical problems resulted in a prolonged surgery time. The other three were excluded because of their extremely fractious or fearful temperament deviation of the protocol.

In the no– TAP group (*n* = 26), 10 Greyhounds, 5 Spanish Hounds, 4 mixed breeds, 2 Beagles, 2 Weimaraners, and one each of Golden Retriever, German Shepherd, and Poodle were included. In the TAP– B group (*n* = 26), 13 Greyhounds, 6 mixed breeds, and one each of Bichon Maltese, Spanish Mastiff, Spanish Hound, Spaniel Breton, Teckel, Weimaraner, and Irish Setter were allocated. The TAP– S (*n* = 20) group included 9 Greyhounds, 5 mixed breeds, 3 Spanish Hounds, 2 German Shepherds, and 1 Pomeranian. Population characteristics and preoperative parameters for each group are shown in Table 1.

There were no significant differences between groups regarding age and weight, surgical time, and pre– or postoperative temperature, but anesthetic time was significantly shorter in the no– TAP group compared to the other groups (Table 1). 7/26 dogs in the no– TAP group, 6/20 in the TAP– S group, and 4/26 in the TAP– B group required a propofol bolus during surgery.

The proportion of dogs that needed rescue analgesia in the 24 h postoperatively was significantly lower for the TAP– B group (9/26) than for the no– TAP (26/26) and TAP– S groups (20/20), but also the proportion of dogs that required analgesia in the first 8 h (6/26 in the TAP– B group, 26/26 in the no– TAP group and 20/20 in the TAP– S group) and within 8 to 24 h postoperatively (3/26 in the TAP– B group, 14/26 in the no– TAP group and 11/20 in the TAP– S group) (P = 0.0001). The total amount of postoperative analgesic doses administered was 42 in the no-TAP, 33 in the TAP-S, and 11 in the TAP-B group. The difference was significant (p = 0.001).

**Table 1:** Demographic variables of the three study groups

	Groups			*p*
	no– TAP	TAP– B	TAP– S	
Age (months)	24 (9– 96)	22 (7– 72)	36 (10– 60)	0.15
Weight (Kg)	18.8 (7– 26.9)	21.1 (3.8– 36.4)	15.35 (4.4– 37)	0.3
Surgical time (min)	31 (22– 46)	29 (23– 39)	31 (24– 47)	0.085
Anesthetic time (min)	58 (46– 69)*	64 (59– 78)	67.5 (60– 79)	0.0001
Tº pre-surgery (ºC)	38.5 (38– 39.2)	38.8 (37– 39.5)	38.4 (38– 39)	0.72
Tº post-surgery (ºC)	36.5 (35.4– 38.2)	37 (34.9– 39)	36.7 (35.9– 37.1)	0.21

no– TAP: inhaled anesthesia, TAP– S: inhaled anesthesia and TAP with saline, TAP– B: inhaled anesthesia and TAP with bupivacaine. Data are shown as median and range (min– max). Asterisk indicates significant differences from the other groups. Tº: temperature

### Blood analytes

There were no significant differences in the baseline WBC, neutrophil, lymphocyte, and platelet values. Over time, significant changes were observed in the postoperative WBC, neutrophil, lymphocyte, and platelet values in all groups. Leucocytes increased significantly at T2 in the three groups and T3 in the no– TAP and TAP– S groups. The rise in the neutrophil count was significant at T2 in the three groups, remaining stable at T3. Lymphocytes decreased significantly at T1 in the three groups, remained stable at T2, and returned to baseline values at T3.

Platelets remained constant over time in the no– TAP group; they decreased significantly at T1 in the dogs of the TAP– B group, remaining stable later, and increased significantly at T2 in the TAP– S group with respect to the early time points.

There were no differences in the blood analytes between the groups at any postoperative time point (Table 2).

**Table 2:** Values of the WBC, neutrophil, lymphocyte, and platelet counts over time

		no– TAP	TAP– B	TAP– S	*p between groups*
WBC (x10³/µl)	T0	7.52 (5.70– 16.50)^a^	9.50 (5.16– 14.82)^a^	9.14 (5.30– 12.26)^a^	0.51
	T1	7.14 (3.92– 14.12)^a^	8.05 (1.13– 13.35)^a^	7.90 (3.25– 13.95)^a^	0.92
	T2	12.62 (6.71– 27.27)^b^	11.34 (3.48– 20.67)^b^	11.17 (3.85– 25.31)^a^	0.40
	T3	14.19 (8.26– 23.97)^c^	13.74 (7.72– 21.44)^b^	15.29 (10.37– 25.85)^c^	0.66
	*p over time*	0.0001	0.0001	0.0001	
Neutrophils (x10³/µl)	T0	5.73 (3.03–9.90)^a^	7.13 (4.12– 15.94)^a^	7.62 (3.69–10.84)^ac^	0.18
	T1	5.25 (1.06 − 10.23)^a^	6.07 (0.90–12.05)^a^	5.89 (1.71– 11.76)^a^	0.57
	T2	11.48 (5.85–20.98)^b^	9.19 (2.96–18.80)^b^	12.65 (4.74–21.54)^b^	0.30
	T3	12.30 (6.88 − 19.11)^b^	10.20 (1.57– 20.20)^b^	10.72 (7.53–16.54)^bc^	0.39
	*p over time*	0.0001	0.0001	0.02	
Lymphocytes (x10³/µl)	T0	1.49 (0.57– 3.20)^a^	1.64 (0.66– 3.80)^a^	2.22 (1.13– 3.28)^a^	0.15
	T1	0.90 (0.12– 5.22)^bc^	0.59 (0.04– 2.06)^b^	0.92 (0.14– 1.66)^b^	0.50
	T2	0.90 (0.11– 2.56)^b^	0.72 (0.15– 2.25)^b^	0.70 (0.36– 1.80)^b^	0.90
	T3	1.32 (0.42– 4.67)^ac^	1.21 (0.30– 2.19)^ab^	1.15 (0.23– 2.51)^ab^	0.64
	*p over time*	0.0001	0.0001	0.003	
Platelets (x10³/µl)	T0	228 (136– 467)^a^	238 (139– 374)^a^	250 (155– 367)^a^	0.31
	T1	209 (121– 573)^a^	196 (125– 402)^b^	241 (148.1– 278)^a^	0.42
	T2	203 (138– 580)^a^	180 (107– 484)^b^	270 (211– 440)^b^	0.06
	T3	190 (128– 378)^a^	180 (120– 275)^b^	242 (135– 340)^a^	0.09
	*p over time*	0.25	0.0001	0.005	

WBC: white blood cell, T0: one hour before the laparoscopic ovariectomy, T1: 2−3 h, T2: 6−8 h, T3: 20−24 h; no– TAP: inhaled anesthesia, TAP– S: inhaled anesthesia and TAP with saline, TAP– B: inhaled anesthesia and TAP with bupivacaine. Data are shown as median and range (max– min). Different superscript lowercase letters in each column indicate significant differences between the different time points (p < 0.05) for that analyte within that group

### Complete blood inflammatory markers

There were no significant differences in baseline NLR, PLR, and SII values among the groups. The three markers significantly increased at T1 in the TAP– B group, then remained stable at T2 and T3. In the no– TAP and TAP– S groups, the NLR and the SII increased significantly at T2, then remained constant at T3 or decreased significantly, as in the case of the NLR in the no– TAP group. PLR increased significantly at T1 in the no– TAP group and T2 in the TAP– S group and diminished significantly at T3 compared to T2.

There were no differences observed between groups in any of the ratios at any control point (Table 3).

**Table 3:** Values of the complete blood inflammatory markers neutrophil– to– lymphocyte ratio (NLR), platelet– to– lymphocyte ratio (PLR), and systemic immune– inflammation index (SII) over time

		no – TAP	TAP– B	TAP– S	*p between groups*	
NLR	T0	3.95 (1.50–9.52)^a^	4.35 (1.49–9.79)^a^	3.30 (2.20–5.82)^a^	0.17	
	T1	6.56 (1.00–39.50)^a^	9.28 (2.39–37.75)^b^	5.76 (3.66–19.92)^a^	0.36	
	T2	17.16 (3.00–50.80)^b^	11.08 (3.50 – 54.50)^b^	15.47 (6.90 – 36.02)^b^	0.80	
	T3	8.20 (2.43– 30.02)^c^	8.23 (2.01 – 17.12)^ab^	8.71 (3.88 – 26.68)^ab^	0.95	
	*p over time*	0.0001	0.0001	0.0001		
PLR	T0	152.21 (56.92–337.86)^a^	144.67 (96.63–360.61)^a^	160.22 (118.27–245.13)^a^	0.70	
	T1	255.42 (23.18– 912)^b^	355.55 (15.47–998.90)^b^	237.04 (124.45 – 508)^ab^	0.62	
	T2	301.50 (55–1105)^b^	250 (90–829)^ab^	401.02 (193–583)^b^	0.21	
	T3	168.90 (43.70 – 723.80)^a^	165.47 (63.01– 400)^a^	162.37 (109.56–432.26)^a^	0.87	
	*p over time*	0.001	0.003	0.009		
SII (× 10^3^)	T0	835.03 (221.43 − 2816.32)^a^	1080.98 (433.04–2291.88)^a^	1186.40 (853.92 – 1724.62)^a^	0.29	
	T1	1594.77 (122.15 – 3697.50)^ac^	1699.10 (62.67–6542.85)^b^	1421.20 (765.39 – 5161.50)^a^	0.65	
	T2	3178 (460.10 – 19056.90)^b^	2158.20 (719.70–10853.70)^b^	4645.68 (2036.70–9680)^b^	0.09	
	T3	1879.80 (496.20 – 7151.20)^bc^	1425.10 (3133.50 – 3470.80)^ab^	1753.62 (1170.08 – 7149.50)^ab^	0.34	
	*p over time*	0.0001	0.0001	0.0001		

T0: before surgery; T1: 2−3 h, T2: 6−8 h (T2), and T3: 20−24 h post– surgery. Data are shown as median and range (max– min) in the three study groups: no– TAP: inhaled anesthesia, TAP– S: inhaled anesthesia and TAP with saline, and TAP– B: inhaled anesthesia and TAP with bupivacaine. Different superscript lowercase letters in each column indicate significant differences between the different time points (p < 0.05) for that marker within that group

## Discussion

The results of the present study show that performing the TAP block for canine LapOV does not increase or decrease the inflammatory response measured through the complete blood– derived inflammatory markers.

In veterinary medicine, locoregional anesthesia techniques have been used together with systemic analgesia over the last few years to obtain a multimodal approach to nociception and decrease the dose and the potential side effects of systemic analgesics and anesthetic drugs (Cima et al. 2020; Morgaz et al. 2021; Viscasillas et al. 2021).

Among the locoregional anesthesia techniques, the TAP has shown good analgesic effects on the post– operatory period in cats (Skouropoulou et al. 2018) and dogs undergoing conventional ovariectomy (Cavaco et al. 2022.) and in dogs undergoing LapOV (Espadas-González et al. 2022; Paolini et al. 2022). However, the influence of the TAP block on the stress response has been little studied.

The studies that relate TAP and any of the complete blood cell count-derived inflammatory markers are very limited and only referred to human patients. Two of them did not find significant differences in NLR between the use of intrathecal analgesia or TAP 24 h after laparoscopic colorectal surgery (Han et al. 2021) or in NLR and PLR between the thoracic epidural analgesia and the four quadrant TAP after cytoreductive surgery in patients with peritoneal carcinomatosis (Cata et al. 2021). However, a third study, showed that post-surgery NLR was significantly lower in women undergoing post-operative cesarean section with spinal anesthesia with TAP block than in those with wound infiltration. (Charlen and Sumartono 2020).

The study of the effects of stress hormones on the key immuno-competent cell populations demonstrated that cortisol strongly decreased lymphocyte numbers and increased counts of neutrophils (Zahorec 2021). Therefore, it was suggested that the lesser increase in the total lymphocyte count and NLR observed in patients receiving spinal anesthesia (Surhonne et al. 2019) might be related to the lower cortisol observed in patients undergoing spinal anesthesia in comparison with those under general anesthesia reported in a different study (Milosavljevic et al. 2014).

Some other studies have observed this phenomenon after the TAP block, reporting lower levels of tumor necrosis factor– alpha (TNF– α) and interleukin (IL)– 1β (Canakci et al. 2021), norepinephrine, epinephrine, cortisol, glucose, IL– 6, and IL– 10 (Liu et al. 2019; Qin et al. 2019) in patients undergoing TAP block with bupivacaine (Canakci et al. 2021) or ropivacaine (Liu et al. 2019) for hernia inguinal repair (Canakci et al. 2021), radical gastrectomy (Liu et al. 2019), or laparoscopic gynecological surgery (Qin et al. 2019) than in the placebo groups of TAP with saline (Canakci et al. 2021) standard general anesthesia (Liu et al. 2019; Qin et al. 2019). However, a study in which the mid– axillary TAP block was performed in patients scheduled for total abdominal hysterectomy failed to find any improvement in the stress response over the following 24 h measured through serum cortisol, plasma metanephrine, and normetanephrine (Ismail et al. 2021). The only study in veterinary medicine analyzing the neuroendocrine response after the TAP block, neither observed differences in cortisol concentration 8 h after ovariectomy between the dogs receiving TAP block with water injection or with bupivacaine. (Cavaco et al. 2022). According to the reasoning set above, that could explain the lack of difference in the complete blood-derived inflammatory markers after LapOV between the three groups in the present study.

Furthermore, although buprenorphine has been shown not to have considerable effects on leukocyte-stimulated cytokine production and does not alter select immune parameters in healthy adult dogs (Monibi et al. 2015), its use for control of acute postoperative pain in a significantly higher number of animals in the TAP– S and no– TAP groups than in the TAP– B group could have also contributed to the lack of difference in the inflammatory markers between the three groups. For the control of the postoperative pain, we followed the protocol of similar studies in dogs and humans that compared TAP-B with TAP-S (Cavaco et al. 2022) or with thoracic epidural (Cata et al. 2021) and used opioids as rescue pain relief in the post-op period. Those studies found significant differences in opioid consumption between the techniques compared in each study but not in the inflammatory markers (cortisol or NLR and PLR) as happened in the present study. A third study analyzing the effects of thoracic epidural or intravenous analgesia on the NLR in thoracotomy cases produced the same result, revealing that NLR was similar at the end of the follow-up period while the additional analgesics requirement between the groups was different (Alkan et al. 2018).

Moreover, as there are no differences in the inflammatory markers and the postoperative opioid requirements between the no-TAP and the TAP-S groups, it can be deduced that the hydrodissection associated with the TAP block technique does not increase the inflammatory response.

This lack of difference between the three study groups could be also attributable to the fact that the LapOV is a routine surgery with low complications that implies a controlled and low pain intensity, which might have been unable to show differences in the complete blood–based inflammatory markers between groups. It has been suggested that the magnitude of the response of peripheral leukocytes would be proportional to the extent of surgical trauma and might be augmented and prolonged when the patient undergoes major surgery (Kim et al. 2011). After other procedures such as femorotibial joint surgery, the inflammatory response measured through the acute phase proteins in dogs was not altered either by the use of pre– emptive extradural bupivacaine and morphine (Sibanda et al. 2006).

In the present study, the pattern changes in NLR, PLR, and SII over time differed among the three groups.

While in the TAP– B group the first significant increase appeared 2−3 h after the surgery, the other two groups exhibited this increase later, at 6−8 h after the surgery. The presence of bupivacaine but not the hydrodissection during the TAP block could be responsible for that early onset of inflammation. Myotoxicity caused by local anesthetic agents is an inevitable concomitant of regional anesthesia techniques (Padera et al. 2008), and bupivacaine– induced myotoxicity is more than obvious than that of lidocaine and ropivacaine (Hussain et al. 2018). However, the values of NLR, PLR, and, especially, SII were lower in the TAP– B group than in the others at 6−8 and 24 h after the surgery, although without significance. This might also be attributable to the immunomodulatory power of the TAP described in human medical studies and the analgesic effects demonstrated in veterinary patients.

The immune response to trauma includes the suppression of cellular immunity by lymphocytes, activation of the inflammatory response characterized by neutrophilia, and increased platelet activation and function (Wang et al. 2021). In the present study, significant increases in WBC and neutrophil counts and a reduction in the lymphocyte count were observed after surgery in the three groups.

Although our results did not find any difference in the lymphocyte count between the TAP– S and TAP– B groups, it has been shown that the count of lymphocytes was higher 4 h after epidural anesthesia with lidocaine added to general anesthesia with propofol compared to the epidural application of a saline solution in dogs undergoing ovariohysterectomy (Imani Rastabi et al. 2021). However, our results agreed with another study that showed a transient decrease in the lymphocyte counts attributable to apoptosis between 3 and 24 h after epidural with lidocaine (Simeonova et al. 2008), as we observed in the present study.

One limitation of the study is the high number of Greyhounds included in each group, which might bias the results due to their unique clinicopathologic characteristics. Nonetheless, we considered that the study sample is representative of the canine population in our country because Greyhounds are popular dogs in Spain. However, the sample heterogenicity concerning breed and age might affect the complete blood inflammatory markers. The longer anesthetic time observed in the TAP– S and TAP– B groups constitutes the second limitation of the study. The impact of this fact on the results is difficult to establish because no study has analyzed the effect of the time under inhaled anesthesia on the blood inflammatory ratios, and an earlier study showed that the combination of the TAP block with inhalational anesthesia for the LapOV reduced the requirements of isoflurane (Espadas-González et al. 2022). Finally, the third limitation could be the low number of dogs, in particular in the TAP– S group, which was the minimum necessary according to the statistical analysis. The presence of a group of TAP with saline was controversial because of the extra harm inflicted on the dogs and because not all similar studies included a sham group. However, the existence of the TAP– S group was considered necessary for the purpose of this study, and the procedure was deemed mild according to the legal classification and was accepted by the ethical committee. Consequently, we decided to keep the number of dogs recruited for that group to a minimum. Although we kept the animals on the statistical analysis after the analgesic rescue, which might have influenced the inflammatory markers, our study design is similar to that of other studies with the same purpose.

To conclude, the present study analyzed for the first time the effect of the TAP on the inflammatory markers NLR, PLR, and SII in dogs undergoing LapOV. The results showed that there is no difference in the inflammatory response triggered after the LapOV measured through the complete blood– derived inflammatory markers when the TAP block with bupivacaine is performed or when the postoperative analgesia is provided exclusively with opioids. The study confirms the contribution of the TAP with bupivacaine to reduce opioid consumption after this surgery. The hydrodissection associated with the TAP block technique does not increase the inflammatory response.

## Data Availability

The datasets generated during and/or analyzed during the current study are available from the corresponding author upon reasonable request.
